# Effects of Forest Gaps on Soil Properties in *Castanopsis kawakamii* Nature Forest

**DOI:** 10.1371/journal.pone.0141203

**Published:** 2015-10-23

**Authors:** Zhongsheng He, Jinfu Liu, Songjin Su, Shiqun Zheng, Daowei Xu, Zeyan Wu, Wei Hong, James Li-Ming Wang

**Affiliations:** 1 College of Forestry, Fujian Agriculture and Forestry University, Fuzhou, Fujian, China; 2 Department of Mathematics, University of Alabama, Tuscaloosa, Alabama, United States of America; Institute for Sustainable Plant Protection, C.N.R., ITALY

## Abstract

The aim of this study is to analyze the effects of forest gaps on the variations of soil properties in *Castanopsis kawakamii* natural forest. Soil physical and chemical properties in various sizes and development stages were studied in *C*. *kawakamii* natural forest gaps. The results showed that forest gaps in various sizes and development stages could improve soil pore space structure and water characteristics, which may effectively promote the water absorbing capacity for plant root growth and play an important role in forest regeneration. Soil pore space structure and water characteristics in small gaps showed more obvious improvements, followed by the medium and large gaps. Soil pore space structure and water characteristics in the later development stage of forest gaps demonstrated more obvious improvements, followed by the early and medium development stages. The contents of hydrolysable N and available K in various sizes and development stages of forest gaps were higher than those of non-gaps, whereas the contents of total N, total P, available P, organic matter, and organic carbon were lower. The contents of total N, hydrolysable N, available K, organic matter, and organic carbon in medium gaps were higher than those of large and small gaps. The disturbance of forest gaps could improve the soils’ physical and chemical properties and increase the population species’ richness, which would provide an ecological basis for the species coexistence in *C*. *kawakamii* natural forest.

## Introduction

Forest gap occurrence is due to internal factors, external factors, or the combined effects of these factors, which lead to the death of dominant trees in the mature stage and subsequently create a gap in the canopy layer of the forest. As an important interference that occurs frequently in the forest, gap disturbance has a close relationship with biodiversity, which is the basis for species’ coexistence in forest communities [1, 2]. It is also an important process for forest regeneration and succession. Gap disturbance promotes the improvement of micro-environmental conditions including solar radiation, air temperature, relative humidity, soil temperature, and soil water content. These conditions affect soil physical and chemical properties which correlate with gap sizes and development stages [3]. The variations of soil properties in forest gaps have a vital role in seed germination, seedlings’ establishment, and recruitment, which affect the regeneration of different plant species and the community structure dynamic [4, 5]. Therefore, a study of soils’ physical and chemical properties in forest gaps can provide a better understanding of the potential capacity of soil supplying nutrients to plant roots. This reference value provides a detailed understanding of the relationship between plants and soil in forest gaps and demonstrates the gap regeneration mechanism.

*Castanopsis kawakamii* Hayata is a valuable and rare plant in the southern forest region of China that distributes sporadically in mountain and hilly evergreen broad-leaved forests of Fujian, Guangdong, Guangxi, and Taiwan [6]. However, above 700 hectares there is an abundance of pure forest. The age of the population is around 100 years in Sanming *C*. *kawakamii* Nature Reserve, Fujian Province. This is a transitional forest type ranging from central to southern subtropical evergreen broad-leaved forests [7, 8]. Therefore, many researchers have launched research on gap regeneration and soil research in this district. The spatial and temporal characteristics of microclimates were heterogeneous in *C*. *kawakamii* natural forest gaps, which led to abundant ecological differentiation, rich variety of forest cover, and species coexistence. This may directly affect the soils’ physical and chemical properties in forest gaps [9]. Meanwhile, many researchers studied soil carbon balance [10] and soil respiration [11] in this natural forest, which helps us to understand the local soil fertility and ecosystem function. However, the effects of gap formation on soil physical and chemical features remains unknown, especially with severe fragmentation in canopy layers of the forest, which limits our understanding of the regeneration pattern of this natural forest. Therefore, the main objective of this study was to observe the effects of forest gaps in different sizes (between 150~500 m^2^) and development stages (early, medium and later) on the soil properties in forest gaps, which could provide a theoretical basis for forest regeneration and population restoration in this *C*. *kawakamii* natural forest.

## Materials and Methods

### Study site and stand history

Sanming *Castanopsis kawakamii* Nature Reserve gave the permission to conduct the study on this site. The authority responsible for a national park, the relevant regulatory body concerned with protection of wildlife, etc. We confirm that the field studies did not involve endangered or protected species.

**Fig 1:**
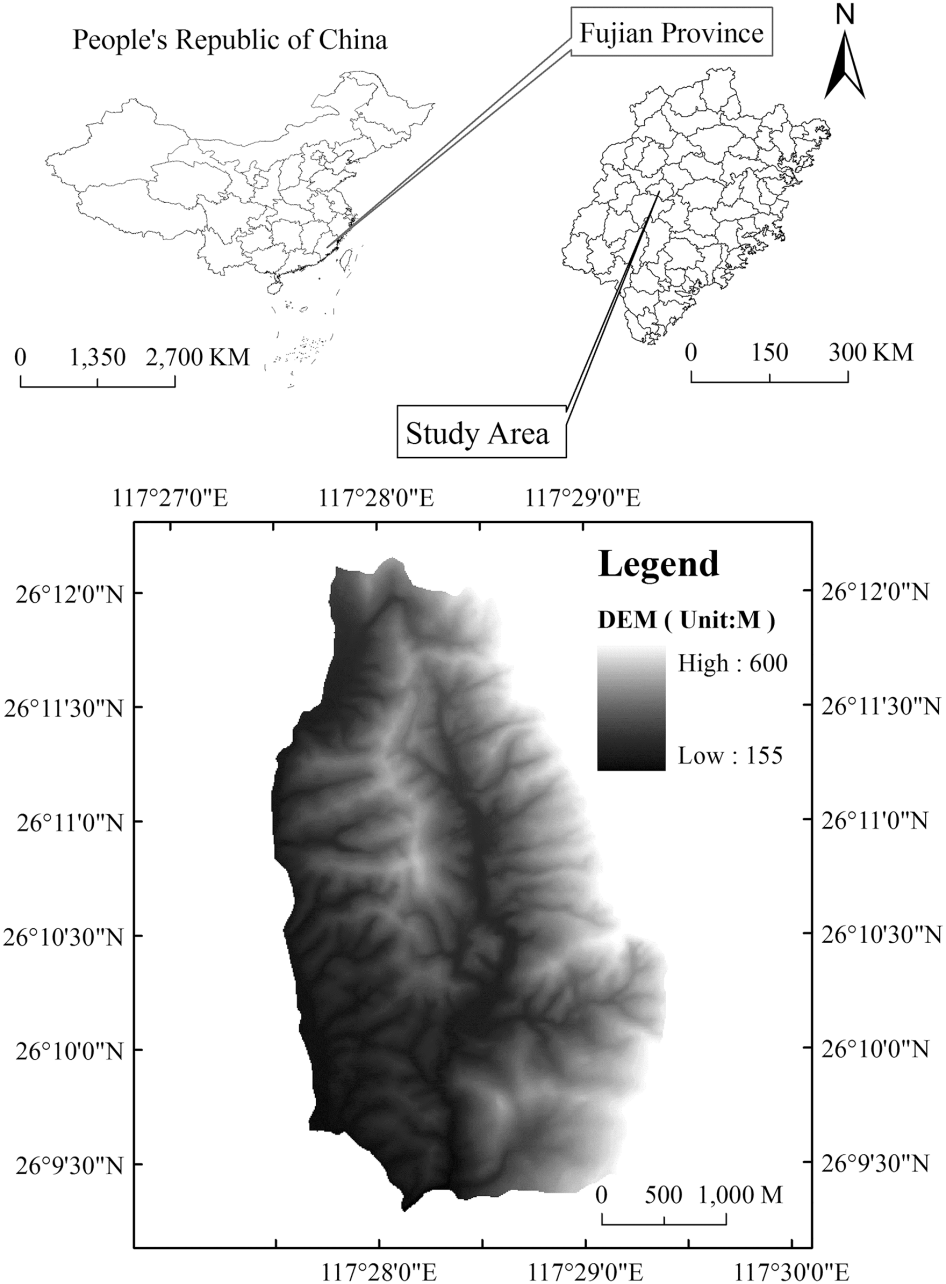
Geographic location of *C*. *kawakamii* nature reserve.

This study site was located in *C*. *kawakamii* Nature Reserve (N26°07'~26°12', E117°24'~117°29') in the middle subtropical area of China. The altitude varied between 180~604 m as shown in Fig 1. With a middle subtropical monsoonal climate, the mean annual temperature is 19.5°C (average of 40 years data by Sanming city Meteorological Bureau), annual precipitation is 1 500 mm, the annual average relative humidity is 79%, and mean velocity of wind is 1.6 m/s, respectively. The soil type in this forest mainly consists of dark-red earth with abundant humus, which is rich in soil nutrition. It is the largest and purest C.kawakamii natural forest in the world, with the canopy closure of about 80% [6]. The main species consisted of *C*. *kawakamii*, *C*. *carlesii*, *C*. *fargesii*, *C*. *eyrei*, *Pinus massoniana*, and *Schima superba*, etc, which formed a unique landscape in subtropical evergreen broad-leaved forest.

### Selection of forest gaps

Based on the previous survey in the permanent sample plots of forest gaps in 2003, we investigated 12 forest gaps according to gap sizes and development stages. Forest gap size was divided by gap area, which was calculated by an oval area or divided into multiple triangles in order to accurately measure them. The oval-shaped areas were calculated by the length axis and short axis. The average gap area of *C*. *kawakamii* natural forest was 327.83 m^2^. We categorized gap area ranging from 100 to 250 m^2^ into small gap, from 250 to 400 m^2^ into medium gap, and the area above 400 m^2^ into large gap. In the present study, 3 small gaps, 6 medium gaps, and 3 large gaps were designed to analyze the effect of gap size on soil properties. Meanwhile, the developmental stages of forest gaps were classified into early, medium, and later stages by the gap formation time, the decomposition degree of gap makers, and species regeneration dynamic in herb and shrub layers [5]. The early stage gap formation occurred within the last 10 years and is mainly composed of strong pioneer species. The medium stage of forest gap consisted of both pioneer species and shadow-tolerant species and its formation age ranged from 10 to 20 years. The gap formation age of the later stage was more than 20 years and species composition mainly consisted of shadow-tolerant species. 3 early stages, 4 medium stages, and 5 later stages of forest gaps were surveyed to study the response of soil properties to the development stages in this natural forest. Meanwhile, we randomly set three non-gap soil samples which were 10 meters away from the gap edge as control groups.

### Soil sample collected and measurement

Three soil profiles from 0 to 30 cm below the soil surface were excavated in each gap. The soil samples were collected by the quarter method. We used the soil core samplers with a capacity of 200 cm^3^ for physical properties collection. Meanwhile, the soils were blended before sealing the plastic bag and transporting them back to lab for chemical properties’ measurement. Accordingly, we randomly set three non-gap soil samples as control groups to compare the differences of soil properties between forest gaps and non-gaps. The measurements of soil properties were based on the reference of Forest Soil Analysis Method [12]. Soil moisture and pore composition were measured by the soil core method including soil bulk density, soil water mass content, soil volumetric moisture content, maximum moisture capacity, capillary water capacity, minimum water-holding capacity, non-capillary porosity, capillary porosity, soil total porosity, and soil aeration degree. Meanwhile, chemical properties of soil pH, total nitrogen (TN), hydrolysable nitrogen (HN), total phosphorus (TP), available phosphorus (AP), available potassium (AK), and soil organic matter (SOM) were measured in each soil plot. Each soil sample was repeated 3 times on average.

### Data processing

Four groups were divided to analyze the significant differences of soil physical and chemical properties for large, medium, small gaps and non-gaps. Also another four groups of early stage, medium stage, later stage and non-gaps were analyzed the significant differences of soil physical and chemical properties. Differences in soil moisture and pore composition, and soil chemical properties of different gap sizes and development stages of forest gaps were analyzed with single-factor analysis of variance (ANONA) and Bonferroni multiple comparisons test. All statistical analyses were performed using the program SPSS 19.0 for Windows.

## Results

### Soil physical and chemical properties in different gap sizes of *C*. *kawakamii* natural forest

The soil water mass content quality and capillary porosity of large and medium gaps were significantly higher than those of small gaps and non-gaps. The soil volumetric moisture content of large gaps was significantly higher than those of medium gaps and non-gaps. The ratio of non-capillary porosity to capillary porosity along with non-capillary porosity of small gaps was significantly higher than those of large, medium, and non-gaps (Table 1). The moisture factors and porosity of the soil composition were increased compared with non-gaps. The values of soil bulk density, soil water mass content, soil volumetric water content, and capillary porosity in large gaps were superior to the medium, small, and non-gaps.

**Table 1:** Soil pore space structure and soil water characteristics in different gap sizes of *C*. *kawakamii* natural forest gaps.

Soil physical properties	Large gaps	Medium gaps	Small gaps	Non-gaps
**Soil bulk density (g·cm** ^**-3**^ **)**	1.33±0.21a	1.27±0.18a	1.22±0.09a	1.31±0.21a
**Soil water mass content (g·kg** ^**-1**^ **)**	282.90±73.00a	246.23±82.17a	179.06±31.43b	205.43±69.61b
**Soil volumetric moisture content (g·kg** ^**-1**^ **)**	359.56±105.60a	296.90±66.59b	343.79±30.99ab	298.88±76.26b
**Maximum moisture capacity (g·kg** ^**-1**^ **)**	383.34±96.94a	396.87±80.52a	418.50±33.62a	379.49±99.63a
**Capillary water capacity (g·kg** ^**-1**^ **)**	332.82±63.98a	345.28±67.15a	321.99±18.88a	312.03±65.78a
**Minimum water-holding capacity (g·kg** ^**-1**^ **)**	270.37±77.51a	238.85±64.12a	281.37±22.58a	235.10±71.36a
**Non-Capillary Porosity (%)**	5.85±5.60b	6.13±4.06b	11.64±2.58a	8.09±4.24b
**Capillary porosity (%)**	43.54±6.38a	43.04±5.42a	39.36±3.01b	39.66±3.60b
**Non-Capillary Porosity/ Capillary porosity**	0.14±0.16b	0.15±0.11b	0.30±0.08a	0.21±0.12b
**Soil total porosity (%)**	49.39±6.26a	49.17±4.61a	51.00±2.44a	47.75±5.06a
**Soil aeration porosity (%)**	13.44±10.20a	19.48±6.01a	16.63±3.02a	17.86±7.39a

Data (mean± SD) marked with different letters(a, b) in plots were significantly different under *p* level as 0.05 in the same row.

The soil chemical properties in different gap sizes of *C*. *kawakamii* natural forest are shown in Table 2. The pH value and C/N ratio in large gaps is higher than those of non-gaps. The concentration of total N in non-gaps is higher than that of forest gaps, while the concentration of hydrolysable N in non-gaps is lower than those of forest gaps, which indicated that forest gaps could improve the convention rate from inorganic nitrogen to organic nitrogen. Meanwhile, the contents of total P and available P in non-gaps are superior to those of forest gaps, which demonstrate that the soil in forest gaps lacks enough phosphorus to promote plant growth. With the increasing size of forest gaps, the contents of total K and available K also increased, while those in non-gaps were relatively low. The contents of organic matter and organic carbon in non-gaps were the highest, while the hydrolysable N and available K were the lowest among various gap sizes.

**Table 2:** Soil chemical characteristics in different gap sizes of *C*. *kawakamii* natural forest gaps.

Soil chemical properties	Large gaps	Medium gaps	Small gaps	Non-gaps
**pH value**	4.43±0.32a	4.24±0.20a	4.30±0.26a	4.18±0.20a
**Total N/g·kg** ^**-1**^	0.77±0.20b	1.22±0.32ab	1.02±0.55ab	1.30±0.41a
**Hydrolysable N/mg·kg** ^**-1**^	106.87±33.43ab	120.12±22.87a	108.12±22.62ab	89.65±22.04b
**Total P/g·kg** ^**-1**^	0.16±0.02b	0.19±0.05ab	0.20±0.04ab	0.23±0.05a
**Available P/mg·kg** ^**-1**^	3.46±0.29b	3.86±1.73b	4.06±1.98ab	5.91±1.48a
**Total K/g·kg** ^**-1**^	41.59±8.03a	31.93±9.52a	28.14±13.40a	30.85±9.80a
**Available K/mg·kg** ^**-1**^	76.14±2.90ab	80.26±22.62a	75.67±19.29ab	61.18±13.23b
**Organic matter/g·kg** ^**-1**^	30.42±8.92ab	34.93±4.68ab	25.98±7.92b	38.14±9.29a
**Organic carbon/g·kg** ^**-1**^	17.65±5.17ab	20.26±2.72ab	15.70±8.68b	21.89±5.10a
**C/N**	25.10±13.59a	17.04±2.94a	16.51±5.19a	17.78±5.47a

Data (mean± SD) marked with different letters(a, b) in plots were significantly different under *p* level as 0.05 in the same row.

### Soil physical and chemical properties in different development stages of *C*. *kawakamii* natural forest gaps

The different developmental stages of forest gaps could effectively improve soil moisture characteristics and porosity composition, in particular, with the early stage and later stage (Table 3). The values of maximum moisture capacity, capillary water capacity, capillary porosity, soil total porosity, and soil aeration porosity in the early stage of forest gaps were higher than those of the medium and later stages of forest gaps and non-gaps. However, the values of soil water mass content, minimum water-holding capacity, non-capillary porosity, and non-capillary porosity/capillary porosity ratio were superior to those of the early and medium stages of forest gaps and non-gaps. According to the significant difference tests, the contents of soil aeration porosity in the early stage of forest gaps were significantly higher than those of medium gaps, while soil bulk density was lower than those of medium stage gaps. The content of capillary water capacity was higher in early stage of forest gaps than those of non-gaps. Moreover, the values of soil volumetric moisture content in the medium stage of forest gaps were higher than those of non-gaps. However, no significant differences of certain soil physical factors were found in different stages of forest gaps and non-gaps such as soil water mass content, maximum moisture capacity, minimum water-holding capacity, and the ratio of non-capillary porosity/capillary porosity.

**Table 3:** Soil pore space structure and soil water characteristics in different development stages of *C*. *kawakamii* natural forest gaps.

Soil physical properties	Early stage	Medium stage	Later stage	Non-gaps
**Soil bulk density(g·cm** ^**-3**^ **)**	1.20±0.14b	1.36±0.17a	1.25±0.17ab	1.31±0.21ab
**Soil water mass content(g·kg** ^**-1**^ **)**	242.73±88.23a	216.34±73.31a	263.34±75.97a	205.43±69.61a
**Soil volumetric moisture content(g·kg** ^**-1**^ **)**	299.20±56.55ab	343.12±79.72a	324.27±82.34ab	298.88±76.26b
**Maximum moisture capacity(g·kg** ^**-1**^ **)**	428.51±70.20a	361.97±70.78a	410.66±75.25a	379.49±99.63a
**Capillary water capacity(g·kg** ^**-1**^ **)**	359.57±68.49a	314.17±38.22ab	340.15±60.41ab	312.03±65.78b
**Minimum water-holding capacity(g·kg** ^**-1**^ **)**	253.26±57.61a	251.34±46.07a	264.64±77.29a	235.10±71.36a
**Non-Capillary Porosity(%)**	8.00±4.03a	5.80±5.29a	8.41±4.71a	8.09±4.24a
**Capillary porosity(%)**	42.60±6.27a	42.34±5.13a	41.95±5.29a	39.66±3.60a
**Non-Capillary Porosity/ Capillary porosity**	0.20±0.11a	0.15±0.15a	0.21±0.13a	0.21±0.12a
**Soil total porosity(%)**	50.60±4.61a	48.14±4.20a	50.36±4.92a	47.75±5.06a
**Soil aeration porosity(%)**	20.68±4.27a	13.83±8.70b	17.94±6.06ab	17.86±7.39ab

Data (mean± SD) marked with different letters(a, b) in plots were significantly different under *p* level as 0.05 in the same row.

The concentrations of soil hydrolysable N in different stages of forest gaps were higher than those of non-gaps, which indicated that gap soil was rich in organic nitrogen that could promote the growth of plants (Table 4). However, the contents of total N, total P, available P, organic matter, and organic carbon in non-gaps were superior to those of forest gaps in different stages, which presented a similar trend in different gap sizes. The values of pH, total K, available K, and C/N ratio in medium stage gaps were higher than those of early and later stages of forest gaps, which demonstrated that medium stage gaps could maintain soil fertilization. According to the significant difference tests, there were no significant differences among the concentration of total K and C/N ratio in different stages of forest gaps and non-gaps. The values of pH and concentrations of available K in the medium development stage of forest gaps were significantly higher than those of non-gaps, while the concentrations of total N, total P, and available P were lower than those of non-gaps. The concentrations of hydrolysable N and available K in later stages of forest gaps were significantly higher than those of non-gaps, while the concentrations of available P, organic matter, and organic carbon were less than those of non-gaps.

**Table 4:** Soil chemical characteristics in different development stages of *C*. *kawakamii* natural forest gaps.

Soil chemical properties	Early stage	Medium stage	Later stage	Non-gaps
**pH value**	4.16±0.27ab	4.44±0.29a	4.28±0.11ab	4.18±0.20b
**Total N/g·kg** ^**-1**^	1.19±0.21ab	0.99±0.38b	1.04±0.50ab	1.30±0.41a
**Hydrolysable N/mg·kg** ^**-1**^	116.01±17.57a	111.31±26.77ab	114.48±29.77a	89.65±22.04b
**Total P/g·kg** ^**-1**^	0.21±0.06ab	0.16±0.01b	0.20±0.03ab	0.23±0.05a
**Available P/mg·kg** ^**-1**^	4.39±1.69ab	3.95±0.98b	3.35±1.79b	5.91±1.48a
**Total K/g·kg** ^**-1**^	25.93±11.70a	37.72±9.17a	34.43±10.78a	30.85±9.80a
**Available K/mg·kg** ^**-1**^	57.95±2.33b	86.45±13.89a	83.47±16.91a	61.18±13.23b
**Organic matter/g·kg** ^**-1**^	34.51±2.24ab	32.57±7.52ab	29.00±8.90b	38.14±9.29a
**Organic carbon/g·kg** ^**-1**^	21.68±3.26ab	18.89±4.36ab	16.20±6.12b	21.89±5.10a
**C/N**	18.53±3.56a	22.28±12.67a	16.47±3.03a	17.78±5.47a

Data (mean± SD) marked with different letters(a, b) in plots were significantly different under *p* level as 0.05 in the same row.

## Discussions

Gap formation enhances the heterogeneity of micro-environmental factors [4]. Meanwhile, gap area and developmental stages are also important factors in determining soil properties, which can consequently change the variations of soil physical and chemical properties, respiration, microbial activity, and enzyme activity. Moreover, it also could lead the variations of the soil pore composition, soil water balance, and nutrition cycle, which could directly or indirectly affect plant growth and regeneration [13].

The different gap sizes and development stages of forest gaps can effectively improve soil moisture and pore composition, which consequently develop soil water retention capacity and water absorption for plants [14]. The small gaps demonstrated an obvious improvement in soil moisture and porosity composition, followed by medium and large gaps. The turnover time of large gaps was longer than that of medium and small gaps. This is due to high density and relative low depth of litter in soil surface, which led to the decline of soil aeration in large gaps. However, it was in a comparatively stable stage in small gaps with relative low disturbance.

The later development stage of forest gaps can apparently improve soil porosity composition and moisture, followed by the early and medium stages. During the early stage of forest gaps, forest disturbance affected the moisture of soil pore composition, but less quickly than solar light and temperature, which is an indirect and long-term process. The species and ratio of crow inclination of gap border trees [15], fine root distribution, litter thickness, micro-topography, and climate factors may also affect spatial characteristics of soil moisture in forest gaps. The medium stage of forest gaps illustrated an increasing trend in the contents of soil compaction and soil volumetric moisture content due to the adaptation to the gap disturbance and species composition. Meanwhile, it was a better condition for soil aeration due to the higher vegetation density and richness in biodiversity in the later stage of forest gaps. However, the soil water absorption in non-gaps was less than in forest gaps as a result of interception from trees in the canopy layer. This declined the water permeability, strengthened the soil mechanical resistance, inhibited the root growth, and finally limited the improvement of plant growth and regeneration in non-gaps. Our result testified the hypothesis that forest gaps create opportunities for the optimum growth of plant species [16].

The contents of hydrolysable N and available K in different gap sizes and development stages of forest gaps were higher than those of forest non-gaps, whereas the contents of total N, total P, available P, organic matter, and organic carbon were lower than those of forest non-gaps. Large gaps could effectively increase the contents of soil pH, total K, and the ratio of carbon to nitrogen (C/N), while the contents of total N, hydrolysable N, available K, organic matter, and organic carbon in medium gaps were higher than those of large and small gaps. The contents of total N, hydrolysable N, total P, available P, organic matter, and organic carbon in early stage were higher than those of medium and later stage of forest gaps, while the contents of total N, hydrolysable N, total P, available P, organic matter, and organic carbon in medium stage were higher than those of early and later stages of forest gaps. Gap makers decreased the nutrition absorption after the formation of forest gaps [17]. Meanwhile, microbial activity in forest gaps increased the amount of organic matter and promoted the release of total N in soil nutrition due to the decomposition of gap makers and microenvironment heterogeneity. This led to a decreased concentration in soil total N, total P, organic matter, and organic carbon. Consequently it could develop the soil acidic environment and available nutrition supplements in different gap sizes and development stages of forest gaps. Therefore, species richness was relatively high in tree and shrub layer of forest gaps [18]. Moreover, the phosphorus compound in soil was less liable to be absorbed by plants due to metal ions such as Fe^3+^and Al^3+^ in the southern forest soil. This would dictate that the available phosphorus be fixed [19]. Moreover, the reduction of litter in forest gaps would lead to the declination of the contents in soil total P and available P in forest gaps, in accordance with the results in a subtropical humid forest [20].

The effect of forest gaps on soil physical and chemical properties is a complex process [21]. The variations of soil properties are not only related to the gap sizes and development stages, but also related to the litter thickness and its decomposition rate, the return of root biomass, and other factors [22]. The litter decomposition in various sizes of *C*. *kawakamii* natural forest gaps showed that litter loss rates were relatively high in non-gaps and small gaps, while large gaps could significantly decrease the microbial activity and litter decomposition rate. However, the medium gaps with a diameter of about 15 m played a decisive role in the soil nutrition release during the process of litter decomposition [23]. Therefore, *C*. *kawakamii* natural forest gaps could improve soil physical and chemical properties and increase the population species richness, which could provide an ecological basis for the species coexistence and regeneration.

## Conclusions

Forest gaps in various sizes and development stages could improve soil pore space structure and water characteristics (Tables 1 and 3). Soil pore space structure and water characteristics in small gaps showed more obvious improvements, followed by the medium and large gaps. Soil pore space structure and water characteristics in the later development stage of forest gaps demonstrated more obvious improvements, followed by the early and medium development stages. The contents of hydrolysable N and available K in various sizes and development stages of forest gaps were higher than those of non-gaps, whereas the contents of total N, total P, available P, organic matter, and organic carbon were lower (Tables 2 and 4). The disturbance of forest gaps could improve the soil physical and chemical properties and increase the population species’ richness, which would provide an ecological basis for the species coexistence in *C*. *kawakamii* natural forest.
